# Chronic Pain in Latin America: Prevalence, Risk Factors, and Their Association with Dementia

**DOI:** 10.1002/alz.095604

**Published:** 2025-01-09

**Authors:** Hernan Hernandez, Ariel Caviedes, Lorena Oyanedel, Teresita Ramos Franco, Loreto Olavarria, Agustin Ibañez, Hernando Santamaria‐Garcia, Nilton Custodio, Rosa Montesinos, Martin Alejandro Bruno, Andrea Slachevsky, Carolina Ochoa‐Rosales, Claudia Duran‐Aniotz, Carolina Gonzalez‐Silva

**Affiliations:** ^1^ Latin American Institute for Brain Health (BrainLat), Universidad Adolfo Ibañez, Santiago Chile; ^2^ Pontificia Universidad Católica de Chile, Santiago Chile; ^3^ Hospital del Salvador, Santiago Chile; ^4^ Latin American Brain Health Institute (BrainLat), Universidad Adolfo Ibanez, Santiago Chile; ^5^ Global Brain Health Institute (GBHI), Trinity College Dublin (TCD), Dublin Ireland; ^6^ Latin American Brain Health (BrainLat) Institute, Universidad Adolfo Ibáñez, Peñalolén, Santiago Chile; ^7^ Pontificia Universidad Javeriana, Bogotá Colombia; ^8^ Global Brain Health Institute, San Francisco, CA USA; ^9^ Instituto Peruano de Neurociencias, Lima, Lima Peru; ^10^ Research unit, Instituto Peruano de Neurociencias, Lima Peru; ^11^ Instituto de Ciencias Biomédicas (ICBM) Facultad de Ciencias Médicas, Universidad Catoóica de Cuyo, San Juan Argentina; ^12^ Memory and Neuropsychiatric Clinic (CMYN), Neurology Service, Hospital del Salvador and Faculty of Medicine, Universidad de Chile, Santiago Chile; ^13^ Latin American Brain Health Institute (BrainLat), Universidad Adolfo Ibañez, Santiago Chile

## Abstract

**Background:**

Chronic pain (CP) is defined as the persistence of pain beyond the expected recovery period of an injury, or alternatively, with a duration exceeding three months. *It has been recognized as a risk factor for dementia in European and North American cohorts*. However, in Latin America (LA), there remains a significant gap in understanding CP prevalence, its risk factors, and its association with Alzheimer’s disease (AD) and frontotemporal dementia (FTD). *Our study aims to shed light on the impact of CP on the LA population, along with its implications for AD and FTD*.

**Method:**

We analyzed data from the National Health Survey (NHS) conducted in Chile in 2009‐2010 and 2016‐2017. This study comprised 2179 subjects from the 2009‐2010 survey and 1447 subjects from the 2016‐2017 survey. Additionally, we assessed CP in LA patients with dementia (AD and FTD) using a concise 6‐question survey, which evaluated the presence, duration, frequency, location, and intensity of pain. Our patients are from Chile, Peru, Argentina, and Colombia, all of whom actively participate in the Multicenter Consortium to Expand Dementia Research in Latin America (ReDLat).

**Result:**

Our analysis revealed from the Chilean NHS that the prevalence of CP increased by approximately 30% across both surveys among individuals aged 20 to 70. Key factors associated with CP in the Chilean population include reduced mobility, anxiety, depression, malnutrition, socioeconomic disadvantage, and lower educational attainment. *The results also suggest an association between CP and the presence of dementia in the Chilean population* that warrants further detailed investigation. In the analysis of the ReDLat cohort, we observed that *patients with AD report experiencing CP at a higher proportion (53.6%) than patients with FTD (31.3%) and control subjects (34.8%)*.

**Conclusion:**

This study highlights the substantial impact of CP on the LA population, *particularly in AD patients*. To our knowledge, this is the first study in LA to investigate the prevalence and risk factors associated with CP. *Additionally, our findings suggest that CP is associated with the pathology of dementia, emphasizing the critical need for further research in this area*.

